# Lesion-Induced Alterations in Astrocyte Glutamate Transporter Expression and Function in the Hippocampus

**DOI:** 10.1155/2013/893605

**Published:** 2013-09-03

**Authors:** Alexandra E. Schreiner, Eric Berlinger, Julia Langer, Karl W. Kafitz, Christine R. Rose

**Affiliations:** Institute of Neurobiology, Heinrich Heine University Duesseldorf, Universitaetsstraße 1, Building 26.02.00, 40225 Duesseldorf, Germany

## Abstract

Astrocytes express the sodium-dependent glutamate transporters GLAST and GLT-1, which are critical to maintain low extracellular glutamate concentrations. Here, we analyzed changes in their expression and function following a mechanical lesion in the CA1 area of organotypic hippocampal slices. 6-7 days after lesion, a glial scar had formed along the injury site, containing strongly activated astrocytes with increased GFAP and S100**β** immunoreactivity, enlarged somata, and reduced capability for uptake of SR101. Astrocytes in the scar's periphery were swollen as well, but showed only moderate upregulation of GFAP and S100**β** and efficiently took up SR101. In the scar, clusters of GLT-1 and GLAST immunoreactivity colocalized with GFAP-positive fibers. Apart from these, GLT-1 immunoreactivity declined with increasing distance from the scar, whereas GLAST expression appeared largely uniform. Sodium imaging in reactive astrocytes indicated that glutamate uptake was strongly reduced in the scar but maintained in the periphery. Our results thus show that moderately reactive astrocytes in the lesion periphery maintain overall glutamate transporter expression and function. Strongly reactive astrocytes in the scar, however, display clusters of GLAST and GLT-1 immunoreactivity together with reduced glutamate transport activity. This reduction might contribute to increased extracellular glutamate concentrations and promote excitotoxic cell damage at the lesion site.

## 1. Introduction

Glutamate reuptake represents the principal mechanism for inactivation of synaptically released glutamate [1, 2]. In the rodent hippocampus, it is mainly accomplished by astrocytic glutamate transporters (EAATs: excitatory amino acid transporters), namely, GLAST (glutamate/aspartate transporter) and GLT-1 (glutamate-transporter-1; rodent analogues of EAAT1 and EAAT2, resp.; [3–7]). Glutamate uptake is energized by the concomitant inward transport of three sodium ions and a proton, while one potassium ion is transported outward. Consequently, its activation is accompanied by an increase in the intracellular sodium concentration of astrocytes [8, 9].

Under pathological conditions, astrocytes undergo a complex reaction referred to as reactive astrogliosis, which is seen in diverse preparations and conditions ranging from primary cell culture to the intact brain [10, 11]. The hallmarks of reactive gliosis are a massive upregulation of the expression of the intermediate filament *Glial Fibrillary Acidic Protein* (GFAP) and a cellular hypertrophy [12, 13]. Reactive astrocytes display several features of immature astrocytes (e.g., [14, 15]) and can partially reenter the cell cycle [16, 17]. The degree of astrogliosis can vary depending on the specific insult. Following traumatic brain injury, a dense glial scar forms at the lesion site due to strong astrocytic reorganization and proliferation [10, 11, 18]. At some distance to the lesion and scar, astrocytes usually do not divide, and their morphological reorganization and hypertrophy is less severe. The establishment of a glial scar around injured tissue is regarded as hindrance for the growth and regeneration of axons [19, 20]. Notwithstanding, reactive astrocytes might also exert a protective role and support regeneration [11, 13, 21].

There is evidence that reactive gliosis is also accompanied by an alteration in the expression level of glial glutamate transporters [6]. Most studies performed in the rodent and human brain reported an overall downregulation of protein levels of both GLAST and GLT-1 and/or a reduction in functional glutamate uptake in response to brain injury and astrogliosis, respectively (e.g., [22–27]). The reduction in the expression of glial glutamate transporters has been suggested to contribute to the elevation of extracellular glutamate concentrations and to glutamate-mediated excitotoxicity which is observed under many pathological conditions.

Injury-induced changes in the expression of the glutamate transporter subtypes might, however, also depend on the degree of astrogliosis. This might be especially relevant and visible upon a mechanical lesion, where reactivity of astrocytes, as judged for example based on their GFAP expression and morphology, ranges from severe astrogliosis in the scar region to only moderate-mild astrogliosis distant from the lesion [10, 11]. To study this question, we analyzed expression of GLAST and GLT-1 following a mechanical lesion in organotypic tissue slice cultures of the mouse hippocampus. Furthermore, we employed ratiometric sodium imaging as functional assay for glutamate uptake in astrocytes. Our results show that mechanical tissue injury generates subsets of reactive astrocytes depending on the distance from the lesion site, which differ in both morphological features and their ability to take up SR101. Furthermore, these subsets show discrete changes in glutamate transporter expression and glutamate uptake capacity, indicating that glutamate clearance might be largely functional in the periphery of the lesion, but strongly hampered in the scar region.

## 2. Materials and Methods

### 2.1. Ethics Statement

This study was carried out in strict accordance with the institutional guidelines of the Heinrich Heine University Duesseldorf, Germany, as well as the European Community Council Directive (86/609/EEC). All experiments were communicated to and approved by the Animal Welfare Office at the Animal Care and Use Facility of the Heinrich Heine University Duesseldorf, Germany (institutional act number: O52/05). In accordance with the German Animal Welfare Act (Tierschutzgesetz, Articles 4 and 7), no formal additional approval for the postmortem removal of brain tissue was necessary. For generation of acute slices, mice were quickly decapitated (following the recommendation of the European Commission published in: Euthanasia of experimental animals, Luxembourg: Office for Official Publications of the European Communities, 1997; ISBN 92–827-9694-9).

### 2.2. Preparation of Tissue Slice Cultures

Acute tissue slices of the hippocampus were prepared from Balb/c mice (*Mus musculus*) of both genders at postnatal days 7 to 8 (P7-8) using standard procedures. For some experiments, transgenic animals (FVB/N-Tg[GFAPGFP]14Mes/J) expressing green fluorescence protein (GFP) under the GFAP promoter were used (obtained from Jackson Laboratory; Harbor, USA). After decapitation of the animals, brains were quickly excised and hemisected in ice-cold artificial cerebrospinal fluid (ACSF) composed of (in mM) 125 NaCl, 2.5 KCl, 2 CaCl_2_, 1 MgCl_2_, 1.25 NaH_2_PO_4_, 26 NaHCO_3_, and 20 glucose, bubbled with 95% O_2_ and 5% CO_2_, and adjusted to a pH of 7.4. Hemisections were trimmed, and transverse slices (200 **μ**m) comprising the entorhinal cortex, hippocampus, fimbria, and thalamus were prepared using a vibratome (Microm HM650V, Thermo Fischer Scientific, Walldorf, Germany). Slices were transferred to ACSF at 35°C for 30 minutes.

Organotypic hippocampal slice cultures were prepared and cultured according to the protocol introduced by Stoppini et al. [28] with minor modifications. To this end, acute slices were transferred to a Millicell culture insert (PICM ORG 50, hydrophilized PTFE, pore size 0.4 **μ**m; Merck Millipore, Darmstadt, Germany) and maintained at the interface of a serum-based culture medium free of antibiotics in a humidified incubator atmosphere of 5% CO_2_ at 37°C. The culture medium was composed of 30% sterile filtered normal horse serum (NHS; GIBCO/Life Technologies, Darmstadt, Germany), 30% Dulbecco's modified eagle medium (DMEM; GIBCO/Life Technologies, Darmstadt, Germany), and 40% Hank's balanced salt solution (HBSS; GIBCO/Life Technologies, Darmstadt, Germany) supplemented with 38 mM glucose (pH adjusted to 7.3-7.4). The medium was changed three times a week, and the insert surface was washed with medium once a week.

After at least 12 days in culture, a mechanical lesion was performed using a sterile scalpel blade. The lesion was positioned in the CA1 area perpendicular to the *stratum pyramidale*, included the *strata oriens*, *pyramidale*, *radiatum,* and *lacunosum moleculare,* and spanned the entire depth of the slice (cf.  Figure 2). After lesioning, slice cultures were maintained for another 6-7 days (designated as 6-7 days postlesion). Unlesioned control slices were cultured in parallel for a corresponding number of days.

For visualization of cell death, ACSF containing 0.5 **μ**g/mL propidium iodide (PI) was applied to the slice surface and incubated for 3 hours at 37°C and 5% CO_2_, followed by a wash with ACSF. Documentation was either accomplished with an epifluorescence microscope (Nikon Eclipse 90i; Nikon Instruments, Düsseldorf, Germany) or at an Olympus Fluoview300 laser scanning microscope (Olympus, Hamburg, Germany).

Astrocyte soma size in organotypic control and lesioned slices was calculated from images of GFP-fluorescence derived from GFP/GFAP mice which were obtained at a confocal microscope (Olympus Fluoview300; Olympus, Hamburg, Germany; see also below). To this end, a semiquantitative approach was used, in which somata of single cells were manually encircled (cf.  Figure 4(b)), and the resulting area was calculated using ImageJ software (NIH, Bethesda, USA).

All chemicals were purchased from Sigma-Aldrich Chemical (Munich, Germany) unless stated otherwise.

### 2.3. Imaging Experiments

For imaging experiments, organotypic slices were excised from the Millicell inserts and incubated in ACSF containing 2.5 **μ**M SR101 for 30 min at 35°C to stain astrocytes (see above). SR101 is a highly specific and widely used tool for the identification of mature astrocytes in many brain regions including the hippocampus [29, 30]. It has recently been shown to be taken up into mature hippocampal astrocytes via an active transport mechanism involving organic anion transport polypeptides [31]. Wide-field fluorescence imaging was performed using a variable scan digital imaging system (TILL Photonics, Martinsried, Germany) attached to an upright microscope (BX51Wi, Olympus Europe, Hamburg, Germany) and a CCD camera (TILL Imago VGA, Till Photonics, Martinsried, Germany). Images were collected with an Achroplan 40x objective (water immersion, N.A. 0.8, Zeiss, Göttingen, Germany). SR101 was excited at 575 nm, and emission was collected above 590 nm. Excitation wavelength for detection of GFP was 488 nm, and emission was collected above 510 nm.

For intracellular sodium imaging, slices were additionally incubated with the membrane permeable form of the sodium-sensitive fluorescent dye SBFI (SBFI-AM; sodium-binding benzofuran isophthalate-acetoxymethyl ester; Molecular Probes/Life Technologies, Darmstadt, Germany) as described earlier [32–34]. Ratiometric sodium imaging was performed by alternate excitation of SBFI at 340 nm (weakly sodium-sensitive wavelength) and at 380 nm (sodium-sensitive wavelength) at 4 Hz. Emission (>440 nm) was collected in defined regions of interest (ROI) representing cell bodies. Standard dynamic background correction was performed as described earlier [33, 34]. After background correction, the fluorescence ratio (F_340_/F_380_) was calculated for the individual ROIs and analyzed offline using OriginPro 8G Software (OriginLab Corporation, Northampton, MA, USA).

Changes in SBFI fluorescence ratio were expressed as changes in sodium concentration based on *in situ* calibrations as reported before [32, 33, 35]. To equilibrate extra- and intracellular Na^+^-concentrations, SBFI-loaded slices were perfused with saline containing ionophores (3 **μ**M gramicidin D, 100 **μ**M monensin) and the Na^+^/K^+^-ATPase blocker ouabain (100 **μ**M), as well as different concentrations of Na^+^.

The glutamate transporter agonist D-aspartate was applied by a pressure application device (PDES-02D, NPI Electronic GmbH, Tamm, Germany) coupled to standard micropipettes (Hilgenberg, Waldkappel, Germany) placed 20–100 **μ**m from cell bodies of selected cells.

### 2.4. Antibodies

Antibodies employed in this study are listed in Table 1. The following antisera were utilized, which represent well-established, commercially available standard markers: guinea-pig GLT-1 antiserum directed against the C-terminus of rat GLT-1 (Chemicon International/Millipore Corp., Carrigtwohill, Ireland; e.g., [36]) and guinea-pig GLAST antiserum directed against the C-terminus of rat GLAST (Chemicon International/Millipore Corp., Carrigtwohill, Ireland; e.g., [37]). Validation and a detailed characterization of both antibodies were provided before [38–40]. For identification of astrocytes, polyclonal rabbit antibodies against GFAP (GFAP-pAb; Dako Cytomation, Denmark; [40, 41]) and S100*β* (Abcam, United Kingdom; [42]) were employed. When double staining with the latter antibody was performed, a monoclonal mouse antibody detecting GFAP was used (GFAP-mAb; Sigma Aldrich, Germany; [40, 43]).

Standard fluorochrome-conjugated antibodies (AlexaFluor, Invitrogen/Life Technologies, Darmstadt, Germany) were employed as secondary antibodies for immunohistochemistry.

### 2.5. Immunohistochemistry

Prior to immunohistochemical processing, organotypic slices were immersion fixed for 30 min at RT in 4% paraformaldehyde (PFA) in phosphate-buffered saline (PBS) following three washes every 30 min with PBS. Cell membranes were permeabilized, and unspecific binding sites were blocked in PBS containing 0.25% triton-X100 (TX) and 2% normal goat serum (NGS; GIBCO/Life Technologies, Darmstadt, Germany) for 90 min at 4°C followed by incubation with the primary antibody GFAP-pAb (1 : 1000; Dako Cytomation, Glostrup, Denmark), diluted in the same solution over night at 4°C. In case of S100*β*/GFAP double stainings, slices were incubated with a mixture of the primary antibodies GFAP-mAb (1 : 1000, Dako Cytomation, Glostrup, Denmark) and rabbit-S100*β* (1 : 100, Abcam, United Kingdom). After five washes in PBS containing 0.25% TX and 2% NGS, slices were incubated either with guinea pig-anti-GLAST or with guinea pig-anti-GLT-1 (both 1 : 1000, diluted in 0.25% TX/2% NGS/PBS) for 4 hrs at RT. Excess primary antibody was removed with five washes using 2% NGS/PBS. Antirabbit-AlexaFluor594 and antiguinea pig-AlexaFluor488 or antimouse-AlexaFluor488 (1 : 100 in blocking solution) were used for visualization of antibody binding and incubated for 2 hrs at RT. The slices were subjected to DAPI staining (4′,6-diamidino-2-phenylindole; 0.5 **μ**m; Invitrogen), washed three times, and mounted on glass slides with mowiol/DABCO (Calbiochem, Fluka, distributed by Sigma-Aldrich Chemical, Munich, Germany).

Identical conditions were applied to all performed stainings regarding tissue processing and staining procedure. Negative controls were run in parallel to each staining by either omitting all or just one of the primary antibodies. Control stainings in which one of the primary antibodies was omitted showed the identical labeling pattern for the remaining antibody as in the double stainings. Omitting both primary antibodies never resulted in a staining.

Documentation of immunofluorescence was either performed with an epifluorescence microscope (Nikon Eclipse 90i; Nikon Instruments, Düsseldorf, Germany) or a confocal laser scanning microscope (Olympus Fluoview300; Olympus, Hamburg, Germany). The epifluorescence microscope was equipped with a standard DAPI (EX 340–380; DM 400; BA 435–485), FITC (EX 465–495; DM 505; BA 515–555), and TRITC (EX 540/25; DM 365; BA 605/55) filter set. Illumination was provided by an Intensilight fiber lamp (C-GHFI; Nikon Instruments, Düsseldorf, Germany), and emission was detected with a monochrome digital camera (DS-Qi1Mc; Nikon Instruments, Düsseldorf, Germany). Images were collected with either a 20x/0.75 (PlanApoVC, Nikon Instruments, Düsseldorf, Germany) air objective or a 60x/1.40 oil immersion objective (PlanApoVC, Nikon Instruments, Düsseldorf, Germany). NIS-Elements software (Nikon Instruments, Düsseldorf, Germany) was used for image acquisition. For confocal microscopy, an Olympus BX51WI microscope coupled to a confocal laser scanning system (FV300) equipped with a multiline argon (488 nm) and a helium-neon laser (543 nm, both Melles Griot, Bensheim, Germany) was used. Images were collected with either a 20x/0.50 (UMPlanFl, Olympus, Hamburg, Germany), a 40x/0.80 water immersion (LUMPlan, Olympus, Hamburg, Germany), or a 60x/1.40 oil immersion objective (PlanApoVC, Nikon Instruments, Düsseldorf, Germany). A Kalman filter 4 was employed at every scan. Simultaneous or sequential scanning of both fluorophores revealed no difference in their staining patterns, indicating absence of cross-excitation or spectral bleed through. The thickness of *z*-plane sections was 1 **μ**m, and the number of optical sections varied depending on the preparation.

Figures show extended focus images as specified in the figure legends, which were calculated from *z*-stacks of optical sections using ImageJ software (NIH, Bethesda, USA). Images were overlaid employing Adobe Photoshop CS2 (Adobe Systems, Cologne, Germany).

### 2.6. Data Presentation and Statistics

Unless otherwise specified, data are expressed as means ± S.E.M. Data were statistically analyzed by Student's *t*-test employing the procedures implemented in OriginPro 8G Software (OriginLab Corporation, Northampton, MA, USA). A *P* value of <0.05 was considered significant. If not stated otherwise, *n*represents the number of analyzed cells and *N* the number of slices. Each set of experiments was performed on at least three tissue slices obtained from different animals.

## 3. Results

### 3.1. Identification of Astrocytes in Organotypic Slice Cultures

The aim of the present study was to analyze changes in glutamate transporter expression and function in astrocytes in response to a mechanical injury. To this end, we prepared organotypic tissue slice cultures of the mouse hippocampus, a well-established model system in which the layering and basic cellular organization of the neural network are maintained over time [28]. After 19–25 days in culture, organotypic tissue slices had flattened to a thickness of 40–50 **μ**m and were composed of 3 to 4 cell layers. At this stage, propidium iodide assays detected only few dead cells dispersed throughout the slices (3 ± 0.6 cells per CA1 subfield; 18 ± 3.4 in entire preparation; *N* = 6; data not shown), indicating that the preparation was viable and in a stable condition.

**Figure 1:**
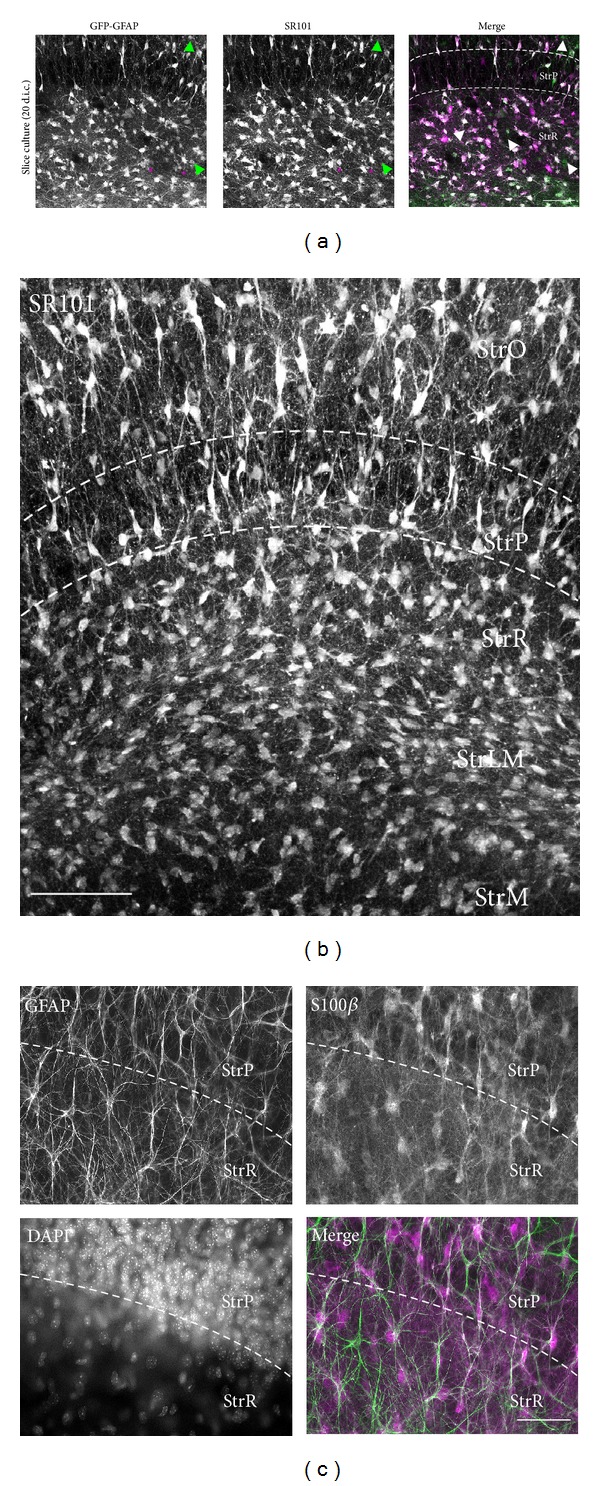
Astrocytes in organotypic hippocampal slice cultures. (a) Organotypic slice cultures at 20 days in culture (d.i.c.). Preparations were derived from animals in which GFP was expressed under control of the GFAP promoter. Shown is the expression of GFP (*left*), the labeling pattern of SR101 (*center*), and the corresponding merged image (GFAP: green, SR101: magenta). Asterisks point out SR101-labeled cells that do not express GFP; arrowheads point out GFP-positive, but SR101-negative cells. Images represent maximum intensity projections of 15 optical sections taken at 1 **μ**m intervals. (b) SR101 fluorescence of an organotypic slice at 21 d.i.c. Image represents a stack of 20 optical sections taken at 1 **μ**m intervals. (c) Immunohistochemical double staining for GFAP and S100*β* as well as DAPI fluorescence and merged image of the fluorescence of GFAP (green) and S100*β* (magenta) of part of the CA1 region encompassing the *strata pyramidale *and *radiatum* of an organotypic slice at 20 d.i.c. Note the radially oriented GFAP-positive fibers crossing the pyramidal cell layer. Images were taken at a wide-field microscope. ((a)–(c)): Dashed lines indicate the approximate boundaries of the *stratum pyramidal*e. StrO, *stratum oriens*; StrP, *stratum pyramidale*; StrR, *stratum radiatum*; StrLM, *stratum lacunosum moleculare*; and StrM, *stratum moleculare*. Scale bars: (a) 50 **μ**m, (b) 100 **μ**m, and (c) 50 **μ**m.

To visualize astrocytes in cultured slices, we used hippocampi of transgenic mice in which green fluorescent protein (GFP) is expressed under the control of the promoter of the astrocyte-specific intermediate filament glial fibrillary acidic protein (GFAP; FVB/N-Tg[GFAPGFP]; “GFP/GFAP mice”). A visualization of the GFP fluorescence demonstrated that the general distribution and organization of astrocytes were preserved in the *strata pyramidale* and *radiatum* of the CA1 region of organotypic cultures, confirming earlier reports (*N* = 3; Figure 1(a); [44, 45]). The vast majority of GFP-expressing cells (~95%) also labeled with the astrocyte-specific vital marker SR101 in organotypic slices (*N* = 3; Figures 1(a) and 1(b)). Furthermore, immunohistochemical stainings for GFAP in slice cultures derived from wildtype animals showed that the majority of GFAP-positive cells were also positive for S100*β* (*N* = 12; Figure 1(c)), a marker for mature astrocytes [46].

Taken together, the nearly complete overlap in the cellular staining pattern for the vital dye SR101 with the expression of GFP in GFP/GFAP mice, as well as the overlap in the immunofluorescence for GFAP and S100*β* in organotypic slices indicates that these markers are well suited to identify astrocytes in this preparation.

### 3.2. Astrocyte Markers and Astrocyte Morphology following Mechanical Lesion

**Figure 2:**
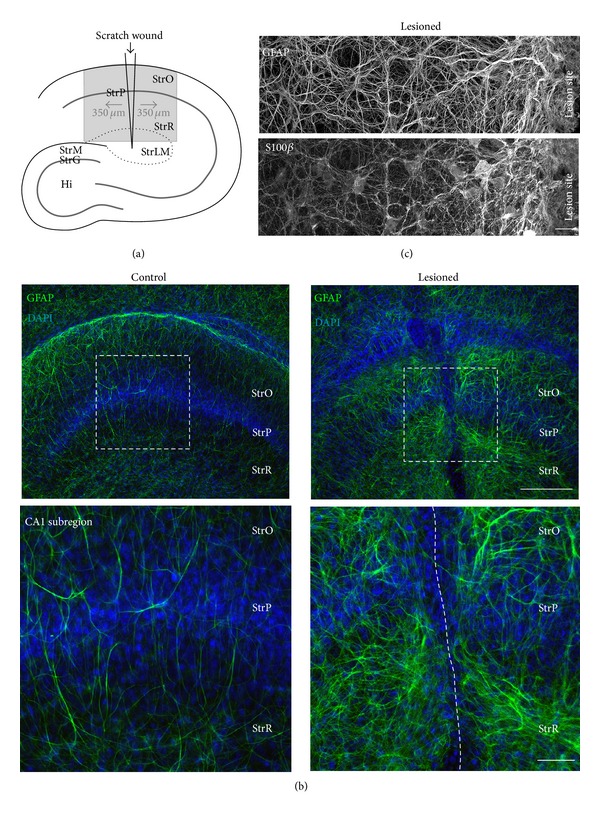
Lesion-induced reactive gliosis in organotypic slice cultures. (a) Schematic overview of the hippocampus indicating the position of the scratch wound. The box delineates the area primarily analyzed. (b) GFAP immunofluorescence and DAPI staining of the CA1 area in a control (*left*) and a lesioned (*right*) slice. The boxes indicate the areas enlarged below. The dashed line in the lower right indicates the lesion. (c) Double staining for GFAP (*top*) and S100*β* (*bottom*) adjacent to the lesion site. Note the gradual increase in GFAP and S100*β* expression towards the lesion. (b): widefield microscopy; (c): confocal microscopy (optical sections taken at 1 **μ**m intervals). StrO, *stratum oriens*; StrP, *stratum pyramidale*; StrR, *stratum radiatum*; and StrLM, *stratum lacunosum moleculare. *Scale bars (b) 200 **μ**m (*top*) and 50 **μ**m (*bottom*); (c) 10 **μ**m.

To induce astrogliosis in organotypic slice cultures, we used a scalpel blade and performed a scratch under semisterile conditions through the entire CA1 region oriented perpendicular to the *stratum pyramidale *(Figure 2(a)). 6-7 days after the lesion, a general enhancement of GFAP immunoreactivity was observed, indicating astrogliosis (Figure 2(b)). The greatest enhancement of GFAP immunoreactivity occurred within a distance of less than 100 **μ**m along the lesion (*N* = 26; Figures 2(b) and 2(c)). Here, a dense meshwork of thick, GFAP-positive labels was present, indicative of the formation of a glial scar. Furthermore, the region close to the lesion site exhibited a discernible increase in S100*β* immunoreactivity (*N* = 12; Figure 2(c)). Both the prominent expressions of GFAP and S100*β* are characteristics of strongly reactive astrocytes, which represent the major cellular component of the glial scar forming after mechanical tissue injury [10]. More distal to the lesion (>100–350 **μ**m), a less dramatic rearrangement of GFAP immunoreactivity was observed.

**Figure 3:**
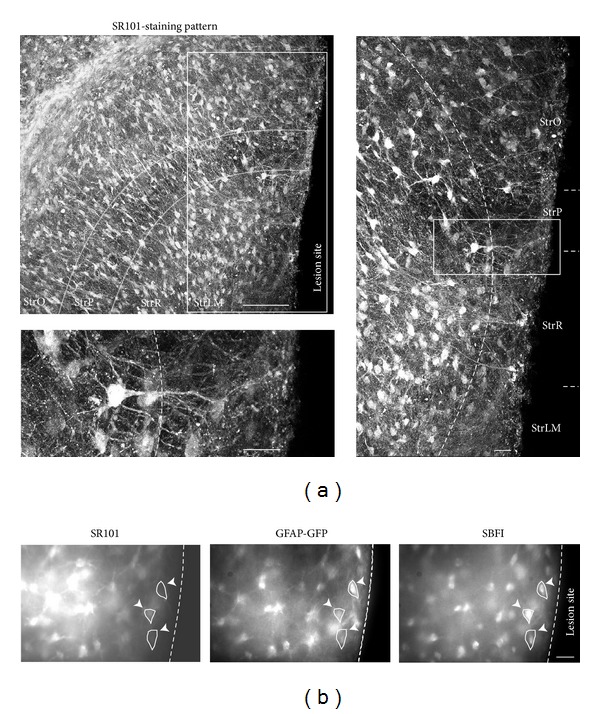
SR101 labeling in lesioned slices. (a) SR101 fluorescence in the CA1 area in a slice 7 days after lesion. The delineated area comprises the *stratum pyramidale*, and the box indicates the area enlarged on the right side. *Right*: SR101 fluorescence at the lesion site, illustrating that SR101 labeling is reduced along the lesion. The dotted line indicates the transition between the scar area and the adjacent, moderately reactive tissue. The boxed area is further enlarged at the bottom. (b) Image series obtained at a wide-field fluorescence microscope. The slice was prepared from a transgenic GFP/GFAP mouse, and the lesion site is indicated by the dotted line. *Left*: Image of SR101 fluorescence, *center:* GFP fluorescence indicating GFAP expression*;* and *right*: SBFI fluorescence. SR101 labeling is only weak in GFP/SBFI-positive cells in the scar region (see arrowheads). StrO, *stratum oriens*; StrP, *stratum pyramidale;* StrR, *stratum radiatum*, and; StrLM, *stratum lacunosum-moleculare*. Scale bars: 100 **μ**m ((a), *upper left*) and 20 **μ**m (other images).

A clear difference in astrocyte properties between the scar region and its periphery was also seen following SR101 labeling. In the periphery of the scar (100–350 **μ**m distance from the lesion), SR101 resulted in a reliable and bright cellular staining, similar to what had been observed in control, unlesioned slices (*N* = 12; Figure 3(a), cf.  Figure 1). In contrast to this, cellular SR101 staining was detectable but only faint within a distance of less than 100 **μ**m on both sides of the lesion (Figure 3(a); mean width of SR101-free area 111 ± 3 **μ**m; *N* = 5). In slices from GFP/GFAP transgenic animals (*N* = 5; Figure 3(b)), GFAP-positive, weakly SR101-positive scar cells also stained with the sodium-sensitive fluorescence indicator SBFI-AM (acetoxymethyl ester of sodium binding benzofuran isophthalate; see below; *N* = 3), indicating that they were able to take up and de-esterify this dye (Figure 3(b)).

In addition to GFAP-positive cells, the scar region hosted microglial cells, identified by their vital staining with Texas Red-coupled lectin from *lycopersicon esculentum* (tomato lectin; *N* = 4; not shown). Tomato lectin-positive microglia, however, did not take up SBFI-AM (*N* = 4; not shown), confirming earlier reports that microglial cells do not incorporate AM-ester dyes *in situ* [47].

**Figure 4:**
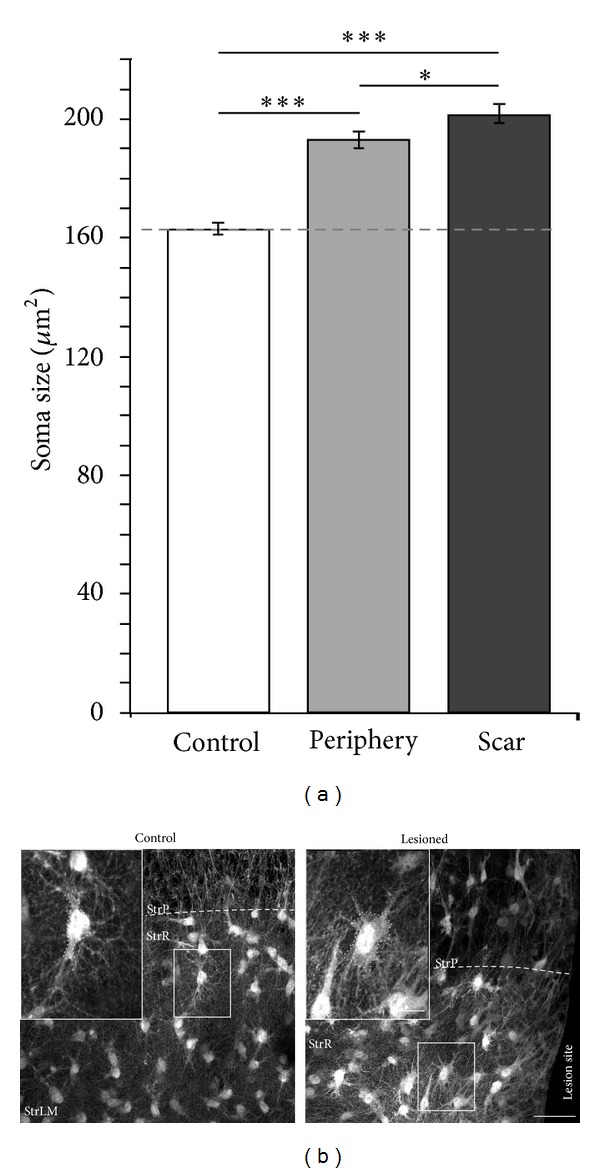
Lesion-induced changes in somatic area. (a) Quantification of somatic area based on GFAP/GFP fluorescence in control and lesioned slices. The soma size increased both in astrocytes in the scar region (“scar”) and in astrocytes in the periphery of the lesion (“periphery”; ***P < 0.001; *P < 0.05). (b) Confocal images of GFP fluorescence in cultured slices obtained from GFP/GFAP transgenic animals under control conditions (*left*) and subjected to a lesion (*right*). Cells surrounded by boxes are shown at higher magnification in the insets, and the dashed lines around the soma illustrate the areas which served to determine their size. StrP, *stratum pyramidale*; StrR, *stratum radiatum*; StrLM, *stratum lacunosum moleculare*; and scale bars: 40 **μ**m and 10 **μ**m (inset).

To further characterize reactive gliosis in response to the scratch wound, we determined the area of astrocyte somata in confocal images of the GFP fluorescence in control and lesioned slices obtained from GFP/GFAP transgenic animals. These measurements revealed that, compared to astrocytes in control slices, the soma area of astrocytes in the periphery of a lesion was increased by 18% and that of astrocytes in the scar region was increased by 24% (control: 163 ± 2 *μ*m^2^; lesion periphery: 193 ± 3 *μ*m^2^; scar tissue: 201 ± 4 *μ*m^2^; *P* < 0.001; *n* = 945, 496, and 439 in 7 control and 9 lesioned slices; Figures 4(a) and 4(b)). Thus, astrocytes show a second hallmark of reactive gliosis after lesion, namely, a hypertrophy of their cell bodies, which is observed both in the scar tissue as well as in the periphery of the lesion.

Taken together, these data show that 6-7 days after the mechanical lesion, a glial scar has formed along the lesion site. This scar encompasses strongly activated astrocytes, characterized by a robust increase in GFAP and S100*β* expression. These proximal reactive astrocytes, located within a distance of less than 100 *μ*m from the lesion, exhibit long GFAP-positive processes, have significantly swollen somata, and only weakly stain with SR101. Astrocytes in the periphery of the scar (100–350 *μ*m from the lesion) display swollen cell bodies as well, but show only moderate upregulation of GFAP and maintain their ability to efficiently take up SR101.

### 3.3. Expression Levels and Spatial Distribution of GLT-1 and GLAST

**Figure 5:**
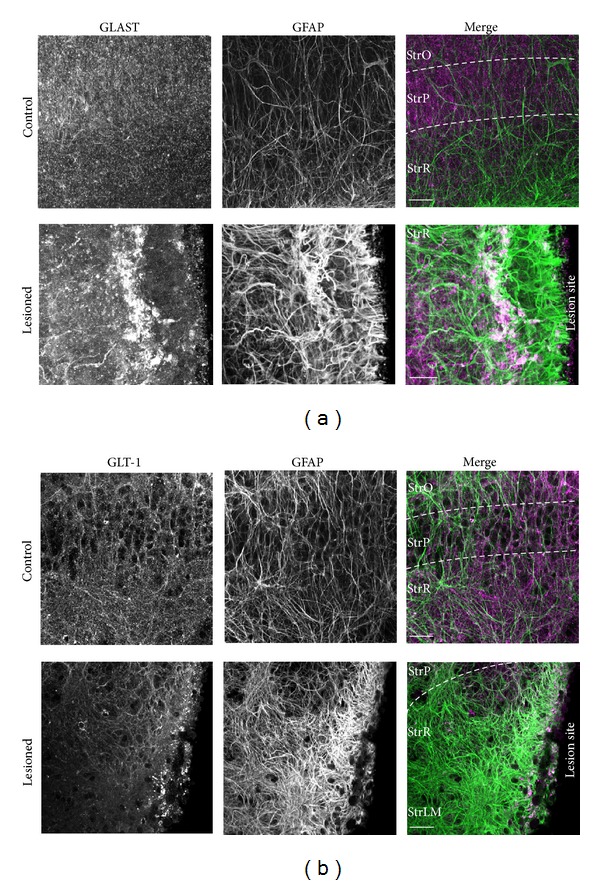
GLAST and GLT-1 immunoreactivity after mechanical injury. (a) Fluorescence images of immunohistochemical stainings detecting the glutamate transporters GLAST (*left*), GFAP (*center*) as counterstaining, and the corresponding merged image (GFAP: green, GLAST: magenta) in control (*top row*) and lesioned slice cultures (*bottom row*). (b) GLT-1 (*left*), GFAP (*center*) double labeling of a control (*top row*) and a mechanically lesioned slice (*bottom row*). The corresponding merge (GFAP: green, GLT-1: magenta) is found on the right. Note that after lesion, glutamate transporter immunoreactive clusters are accumulated in the direct vicinity of the lesion site (arrowheads). GFAP, glial fibrillary acidic protein; GLAST, glutamate aspartate transporter; GLT-1, glutamate-transporter-1; StrO, *stratum oriens*; StrP, *stratum pyramidale*; StrR, *stratum radiatum*; StrLM, *stratum lacunosum-moleculare*; and scale bars: (a) 20 **μ**m, (b) 40 **μ**m.

To determine the spatial distribution of the glutamate transporters GLAST and GLT-1 in organotypic slices and changes therein in response to the lesion, immunohistochemistry was employed. In control slices, GLAST (*N* = 13; Figure 5(a)) as well as GLT-1 immunoreactivity (*N* = 11; Figure 5(b)) appeared as punctate labeling throughout the entire CA1 area. The staining patterns for both transporters in the tissue slice cultures were thus similar to those reported from acute tissue slice preparations of the hippocampus [36, 48, 49].

The relatively homogeneous distribution of immunoreactivity throughout the preparation found in control slice cultures was abandoned following a lesion. Here, distinct accumulations of GLAST (*N* = 17; Figure 5(a)) and GLT-1 immunoreactivity (*N* = 12; Figure 5(b)) were found in the scar region. In both cases, these preferentially colocalized with thick GFAP-positive bundles running in parallel to the lesion (Figure 5). Apart from the marked accumulation of GLAST reactivity in parallel to the scar, GLAST immunoreactivity appeared generally increased in some lesioned slice preparations (Figure 5(a)) but unaltered in others both within the scar as well as in its periphery. For GLT-1, a decline in the intensity of immunoreactivity was observed with increasing distance from the lesion (Figure 5(b)).

In summary, these results reveal distinct alterations in the immunoreactivity of GLAST and GLT-1 following a mechanical lesion. In the scar region, clusters of GLT-1 and GLAST immunoreactivity are present, primarily colocalized with strongly GFAP-positive fibers. Apart from these distinct clusters, GLT-1 immunoreactivity appears to decline with distance from the scar, whereas GLAST immunoreactivity seems largely uniform throughout the preparation.

### 3.4. Sodium Transients Induced by Activation of Glutamate Uptake

**Figure 6:**
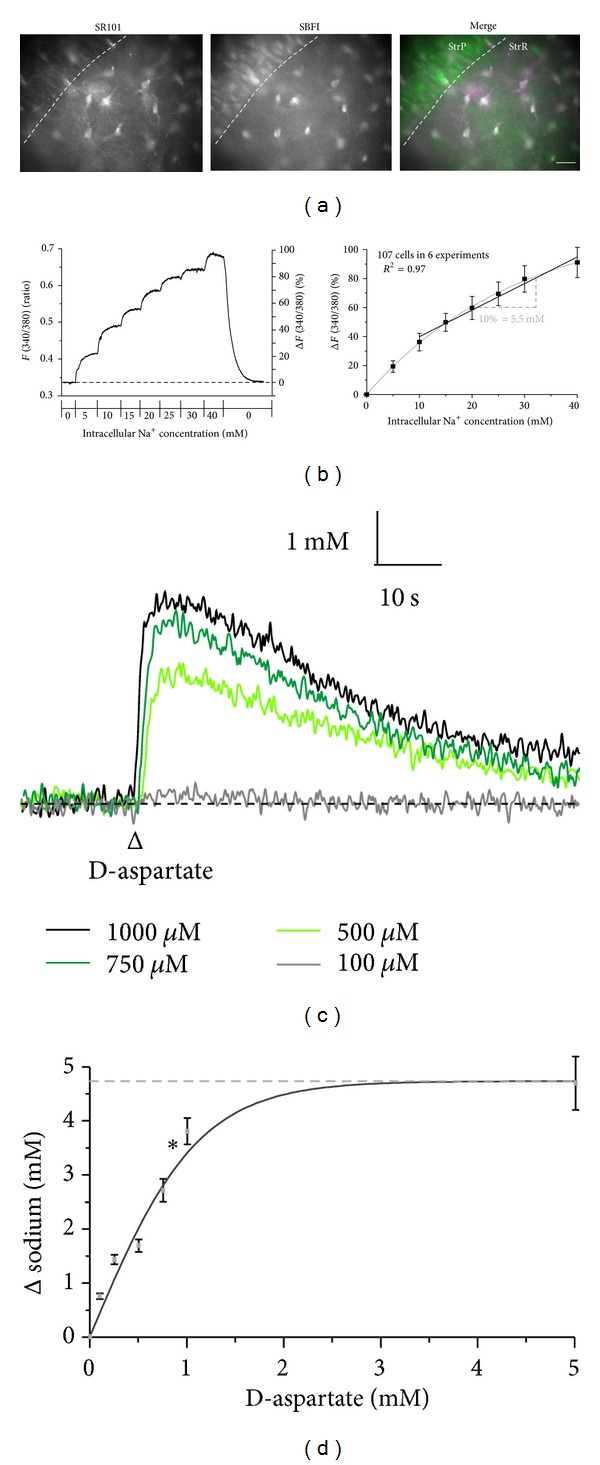
Sodium changes induced by activation of glutamate uptake. (a) Staining of astrocytes by colabeling with SR101 and SBFI. *Left*: epifluorescence image of the CA1-area of an SR101-loaded organotypic slice. *Center*: SBFI fluorescence taken from the same area. *Right*: overlay. SBFI-loaded, SR101-positive astrocytes appear in white (arrowheads). StrP, *stratum pyramidale*; StrR, *stratum radiatum*; scale bar: 20 **μ**m. (b) *Left:* calibration of SBFI fluorescence in an astrocyte. Depicted is the change in intracellular SBFI fluorescence ratio in the calibration solutions containing ionophores and in response to stepwise changes in the sodium concentration. *Right:* mean values ± S.E.M. of the normalized changes in SBFI fluorescence at different sodium concentrations. The black line represents a linear fit of the data points between 10 and 40 mM sodium, illustrating that a 10% change in SBFI fluorescence represents a change in the sodium concentration by 5.5 mM. (c) Sodium transients in a single astrocyte induced by 500 ms applications of D-aspartate at different concentrations. (d) Mean values ± S. E. M. (*n* = 19) of the peak amplitude of sodium transients at different concentrations of D-aspartate. The curve represents a sigmoidal fit of the data.

Glutamate uptake is accompanied by the inward flux of sodium, resulting in increase in the intracellular sodium concentration of astrocytes. To monitor its function, we assayed sodium increases induced by D-aspartate, a nonmetabolized substrate of sodium-dependent glutamate uptake which is also transported [1] in astrocytes in the *stratum radiatum* of organotypic slices. To this end, SR101-stained slices were additionally loaded with the sodium-sensitive fluorophore SBFI-AM (Figure 6(a), cf. Figure 3(b)). *In situ* calibrations of the SBFI fluorescence revealed a linear increase in its fluorescence ratio at intracellular sodium concentrations between 10 and 40 mM (*n* = 107; Figure 6(b)) as reported before [33].

Repetitive pressure application of D-aspartate for 500 ms induced reliable increases in the intracellular sodium concentration in astrocytes, indicating activation of sodium-dependent glutamate uptake (*n* = 488; Figure 6(c)). The experiments were performed in the presence of the sodium-channel blocker TTX (tetrodotoxin, 0.5 *μ*M) to suppress action potential generation. The amplitude of D-aspartate-induced sodium increases was dependent on the concentration applied (Figure 6(c)) and saturated at about 2 mM (*n* = 19; Figure 6(d)), as reported before for mouse cortical astrocytes in culture [50]. At a concentration of 1 mM, the amplitude of D-aspartate-induced sodium transients was near maximum and amounted to 3.8 ± 0.2 mM (*n* = 161; Figures 6(c) and 6(d)), a value comparable to that in astrocytes in acute slices obtained in our lab [33].

**Figure 7:**
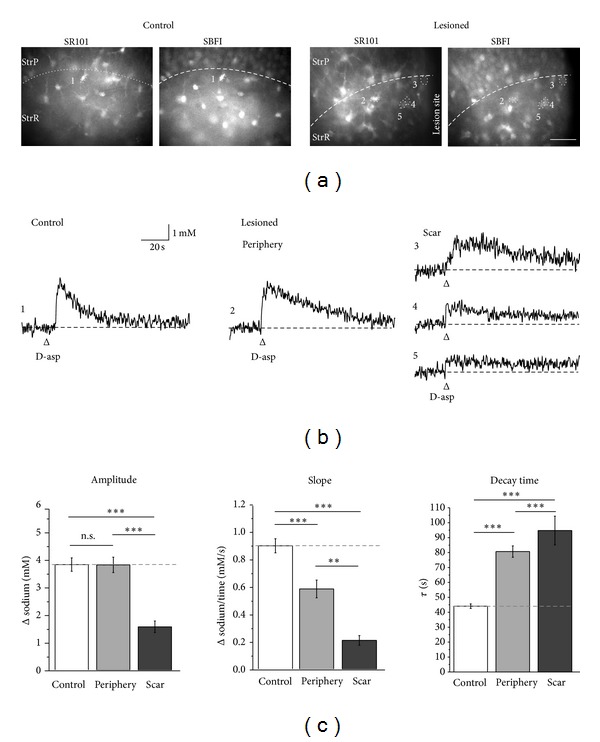
D-aspartate-induced astrocyte sodium transients following lesion. (a) CA1 area of a control (*left*) and a lesioned (*right*) slice preparation. Shown are epifluorescence images of SR101 and SBFI fluorescences. Circled areas and numbers indicate the regions of interest analyzed in the experiment depicted in (b). StrP, *stratum pyramidale*; StrR, *stratum radiatum*; and scale bar: 40 **μ**m. (b) Sodium transients induced by pressure application of 1 mM D-aspartate (D-asp) for 500 ms in the different cells in the control and lesioned slice as indicated in (a). (c) Histograms showing mean values ± S. E. M. of the peak amplitude (*left*), slope (*center*), and decay time (*right*) of D-aspartate-induced sodium transients in astrocytes in control slices (*n* = 161) as well as in cells in the scar region and its periphery (*n* = 111 and *n* = 26, resp.). **P < 0.01; ***P < 0.001. StrP, *stratum pyramidale*; StrR, *stratum radiatum*; and scale bar: 40 **μ**m.

To probe for changes in glutamate uptake, we compared sodium signals induced by 1 mM D-aspartate between control and lesioned slices (Figure 7). In lesioned slices, astrocytes located in the periphery of the lesion (100–350 **μ**m distance) responded with a sodium transient to application of 1 mM D-aspartate (see cell 2 in Figures 7(a) and 7(b)). The amplitude of D-aspartate-induced sodium signals in cells in the lesion periphery was similar to that observed in control slices (3.8 ± 0.3 mM; *n* = 111; see cell 1 in Figures 7(a), 7(b), and 7(c)). Application of D-aspartate also evoked sodium transients in weakly SR101-positive, SBFI-filled astrocytes located in the glial scar area (<100 **μ**m from the lesion; see cells 3–5 in Figures 7(a) and 7(b)). These cells, however, displayed significantly smaller peak amplitudes (1.6 ± 0.21 mM, *n* = 26; *P* < 0.001; Figures 7(b) and 7(c)) than astrocytes in the periphery and in control slices.

The kinetics of D-aspartate-induced sodium transients differed between all three groups. This was true for the slope of the sodium increase (10%–90% change), which was significantly reduced in astrocytes from lesioned slices versus astrocytes from control slices (0.9 ± 0.05 mM/s in control versus 0.6 ± 0.06 mM/s in the periphery and 0.2 ± 0.04 mM/s in the scar region; *n* = 161, 111, and 26, resp.; *P* < 0.001; Figures 7(b) and 7(c)). In addition, the decay back to baseline was significantly slower following lesion (Figures 7(b) and 7(c)). The time needed for the signal to decay by 67% was approximately doubled (decay time *τ* was 44 ± 2 s in control, *τ* = 81 ± 4 s in periphery of the lesion, and *τ* = 95 ± 10 s in scar cells; Figures 7(b) and 7(c)).

These results show that mechanical injury results in distinct alterations in the kinetics and/or amplitudes of D-aspartate-induced intracellular sodium transients in reactive astrocytes, depending on their distance to the lesion.

## 4. Discussion

Our results show that within 6-7 days after setting a scratch wound in organotypic hippocampal slices, a dense glial scar had formed along the lesion site. Up to approximately 100 **μ**m distance from the lesion, the scar tissue was characterized by a substantial elevation of GFAP expression, a pronounced cellular hypertrophy, and reduced uptake of SR101 by astrocytes. While prominent clusters of GLAST and GLT-1 immunoreactivity were observed along GFAP-positive structures in scar cells, D-aspartate-induced intracellular sodium signals were strongly dampened, indicating a significant reduction in glutamate uptake capacity close to the lesion. In the periphery of the lesion (100–350 **μ**m), astrocytes showed less pronounced reactivity, maintained their ability to efficiently take up SR101, and displayed only minor changes in glutamate transporter immunoreactivity and function. Thus, depending on their distance from the lesion, astrocytes showed different grades of reactivity and displayed discrete changes in glutamate transporter immunoreactivity and uptake capacity.

### 4.1. Characteristics of Lesion-Induced Reactive Gliosis in Organotypic Cultures

Organotypic tissue slice cultures of the hippocampus represent a well-established model system, in which the basic tissue architecture is maintained, but which still hosts the major advantages of cell cultures such as good accessibility and control of experimental conditions. They have been used extensively for the analysis of neuronal properties and developing neuronal networks [28, 51, 52] or the study of excitotoxic neuronal damage (e.g., [53, 54]). Recent studies also demonstrated that basic morphological characteristics of astrocytes and the typical glia-synapse organization are well preserved in organotypic slice cultures (e.g., [44, 45, 55, 56]). In addition, we found a nearly complete overlap of SR101 staining with the expression of GFP in slice cultures obtained from GFP/GFAP mice, demonstrating that SR101 is well suited to identify astrocytes in this preparation as described before for acute tissue slices and *in vivo* [29, 30].

Performing a scratch through the CA1 region of organotypic slices resulted in the formation of a glial scar along the lesion site, as judged by the prominent increase in expression of GFAP and S100*β*. In addition, astrocyte cell bodies showed a significant hypertrophy. Furthermore, astrocytes in the periphery of the scar (100–350 **μ**m from the lesion) showed mild to moderate reactivity, displaying swollen cell bodies, and only moderate upregulation of GFAP. These changes are hallmarks of reactive gliosis observed after tissue injury [10, 13, 57]. Generally, the degree of injury and the distance of the astrocytes from the site of injury define the degree of their activation [11, 13].

Our study also revealed that strongly reactive astrocytes in the scar region only weakly stain with SR101. The reduced ability of strongly activated astrocytes to accumulate SR101 indicates that strong glial activation is accompanied by a downregulation of the organic anion transporter responsible for uptake of SR101. This is reminiscent of immature astrocytes in the early postnatal hippocampus, which are SR101-negative [30] and in line with a wealth of experimental evidence suggesting that reactive gliosis represents a process comprising a dedifferentiation of astrocytes [17].

### 4.2. Changes in Glutamate Transporter Expression following Lesion

Glutamate uptake in the hippocampus is mainly achieved by the glial glutamate transporters GLT-1 and GLAST [1, 2]. A central factor in many brain pathologies, including traumatic brain injury, is an increase in extracellular glutamate and excitotoxicity; it has been suggested that a change in the expression levels of glial glutamate transporters might play a critical role in the failure of glutamate clearance [6, 58–60]. Earlier work has found an overall downregulation of protein levels of both GLAST and GLT-1 following astrocyte activation [22–27], indicating that this is causal to the elevation of extracellular glutamate. In contrast to this notion, other studies reported increased glutamate transport capacity of reactive astrocytes and suggested a protective influence [61, 62].

To visualize possible differences in the spatial expression profile of GLT-1 and GLAST in strongly reactive astrocytes along the scar as compared to moderately reactive astrocytes in its periphery, we performed immunohistochemical stainings. These revealed an accumulation of GLT-1 and GLAST immunoreactivity along thick GFAP-positive fibers, which was particularly pronounced for GLAST. Besides these clusters, overall GLAST immunoreactivity seemed unaltered as compared to unlesioned slices, whereas GLT-1 immunoreactivity seemed weaker and declined with increasing distance from the scar. Clustering of glutamate transporters has been described by several reports [48, 63, 64]. In developing hippocampal astrocytes, cluster formation was preferentially found in branches opposed to synapses and was increased with increased neuronal activity, indicating that it is necessary to cope with synaptic release of glutamate [36]. Along the same lines, it was observed that sustained astroglial activation by ciliary neurotrophic factor (CNTF) in the rat striatum induced a concentration of GLAST and GLT-1 into raft microdomains and improved glutamate clearance, indicating that cluster formation increased the cellular capacity for glutamate uptake [61]. However, another study found glutamate-induced clustering of GLT-1 that induced its endocytosis and intracellular trafficking without changing the total expression levels as detected by western blots, arguing for a decrease in functional glial glutamate uptake capacity [49]. In the present study, antibodies against GLT-1 and GLAST were employed for immunohistochemistry after permeabilization of the plasma membrane. The clusters of GLAST and GLT-1 along GFAP-positive fibers in the scar region (as GLAST and GLT-1 immunoreactivity in general) could thus represent glutamate transporters in the plasma membrane or in intracellular compartments or both. Consequently, the observed clustering does not allow a prediction about possible functional changes in glutamate uptake.

### 4.3. Lesion-Induced Changes in Functional Glutamate Uptake Capacity

We probed for the functional activation of glutamate transport by application of the transportable agonist D-aspartate, which results in an increase in the intracellular sodium concentration of astrocytes [8, 9]. The amplitudes of D-aspartate-induced sodium transients in astrocytes in control organotypic slices were similar to those reported from astrocytes in acute slices obtained in our lab [33], indicating similar cellular glutamate transport capacity in both preparations.

Following a lesion, D-aspartate-induced intracellular sodium signals were clearly dampened in scar cells. Moreover, the slope of the increase in sodium was significantly reduced, and the decay back to baseline was slowed. Because D-aspartate was applied at nearly saturating concentration, the reduction in peak amplitudes suggests a reduction in functional glutamate uptake capacity in cells close to the lesion, which might be mediated by a decrease in the overall number of glutamate transporters available at the plasma membrane. In contrast, the alteration in the kinetics of the D-aspartate-induced sodium signals can be explained by the cellular hypertrophy that was observed in reactive astrocytes. At an equal transport-mediated sodium influx across the membrane, the slope of resulting changes in the sodium concentration will be decreased in cells with a larger volume.

Furthermore, the decay of intracellular sodium transients back to baseline is mainly governed by the activity of the Na^+^/K^+^-ATPase as well as by diffusion [8]. The slower recovery is in line with earlier studies reporting a downregulation of the sodium pump following reactive gliosis [65, 66]. Moreover, sodium is not buffered in the cell and travels solely by means of diffusion in the cytoplasm. Hindered diffusion, resulting from increased cytoplasmic protein content or impaired gap junction coupling which both occur upon astrocytic activation, might thus also partially contribute to delayed sodium recovery [12, 34, 67].

In contrast to scar cells, the amplitude of D-aspartate-induced intracellular sodium signals was not significantly altered in moderately activated astrocytes in the periphery of the lesion, indicating that the overall number of functional glutamate transporters was not significantly altered. Because these cells showed hypertrophy as well, the slowed kinetics of the sodium transients are in line with an increased cellular volume as well as a possible downregulation of Na^+^/K^+^-ATPase and/or a slowed diffusion as argued above.

Taken together, our results indicate a significant reduction in glutamate uptake capacity in strongly activated astrocytes in the scar region. Thus, the prominent clustering of GLAST and GLT-1 immunoreactivity along GFAP-positive structures close to the lesion is likely to reflect a loss of glutamate transporters from the plasma membrane upon transporter internalization as reported by Nakagawa et al. [49]. The authors of the latter study speculated that large and prolonged increases in glutamate concentrations are necessary to induce such cluster formation and endocytosis in the tissue, as they would possibly only occur under pathological conditions. Indeed, ambient glutamate concentrations were shown to be significantly increased after traumatic brain injury [68–70]. Along these lines, it can be assumed that in our model system, extracellular glutamate concentrations rose less severely in the periphery of the lesion, preventing a comparable clustering and loss of functional glutamate transport activity in moderately activated astrocytes.

## 5. Conclusions

Our results show complex changes in glutamate transporter expression and function during astrocyte activation following mechanical injury. They confirm that the degree of astrocyte activation depends on the distance from the insult and comprises different endpoints regarding cellular morphology and physiology. Furthermore, our results highlight that astrocytes which show different grades of reactivity also display discrete changes in glutamate transporter expression and function. While immunohistochemistry revealed a prominent clustering of GLT-1 and GLAST immunoreactivity in the scar region, our functional assay clearly showed that glutamate uptake capacity is strongly reduced in scar cells, while it is largely maintained in moderately activated astrocytes in the periphery. Thus, mild-to-moderate astrogliosis in the periphery of a mechanical lesion does not necessarily seem to be accompanied by a significant change in glial glutamate uptake capacity. At the glial scar itself, in strongly reactive astrocytes, a clustering of glutamate transporters is observed that apparently goes along with a severe functional reduction in astroglial glutamate uptake, which may contribute to glutamate-mediated excitotoxicity in this region.

## Figures and Tables

**Table 1:** List of antibodies used.

Antibody	Structure of the immunogen	Company	Order number	Species	Type	Dilution
GLAST (EAAT1)	KPYQLIAQDNEPEKPV ADSETKM	Chemicon/Millipore	AB1782*	Guinea Pig IgG	Polyclonal	1 : 1000
GLT-1 (EAAT2)	AANGKSADCSVEEEPW KREK	Chemicon/Millipore	AB1783	Guinea Pig IgG	Polyclonal	1 : 1000/1 : 2000
GFAP	n.s. (full-length protein from cow spinal cord)	Dako Cytomation	Z 0334	Rabbit IgG	Polyclonal	1 : 1000/1 : 2000
GFAP	clone G-A-5 (LQSLTCDVESLRGTNE SLERQMREQEERHARE AASYQEALTRLEEEGQ SLKDEMARHLQEYQEL LNVKLALDIEIATY)	Sigma-Aldrich	G3893	Mouse IgG	Monoclonal	1 : 1000/1 : 2000
S100ß	n.s. (recombinant full-length cow protein)	Abcam	ab868	Rabbit IgG	Polyclonal	1 : 100
Mouse IgG	AF488 conjugated	Invitrogen (Life Technologies)	A11029	Goat	—	1 : 100
Rabbit IgG	AF594 conjugated	Invitrogen (Life Technologies)	A11012	Goat	—	1 : 100
Guinea pig IgG	AF488 conjugated	Invitrogen (Life Technologies)	A11073	Goat	—	1 : 100

GLAST: glutamate aspartate transporter; GLT-1: glutamate transporter 1; GFAP: glial fibrillary protein; AF: AlexaFluor; appl.: application; IHC: immunohistochemistry; WB: western blot; n.s.: not specified.

*The manufacturer has lately stopped the sale and distribution of the GLAST antibody used here.
